# Overcoming sharp angulation for biliary access with a novel endoscopic retrograde cholangiopancreatography cannula in surgically altered anatomy

**DOI:** 10.1055/a-2762-8067

**Published:** 2026-01-20

**Authors:** Koichi Soga, Mayumi Yamaguchi, Masaru Kuwada, Ryosaku Shirahashi, Ikuhiro Kobori, Masaya Tamano

**Affiliations:** 1 26263 Department of Gastroenterology, Dokkyo Medical University Saitama Medical Center, Koshigaya, Japan

Endoscopic retrograde cholangiopancreatography (ERCP) in patients with surgically altered anatomy (SAA) remains challenging due to the long afferent limb, sharp angulations, and altered biliary-enteric orientation 1 . Selective cannulation of the right intrahepatic bile duct (R-HBD) is particularly difficult because of the steep alignment between the jejunal limb and the R-HBD. Although single-balloon enteroscopy (SBE) has advanced ERCP in postoperative cases, anatomical angulation often limits device maneuverability and cannulation success.

**Fig. 1:**
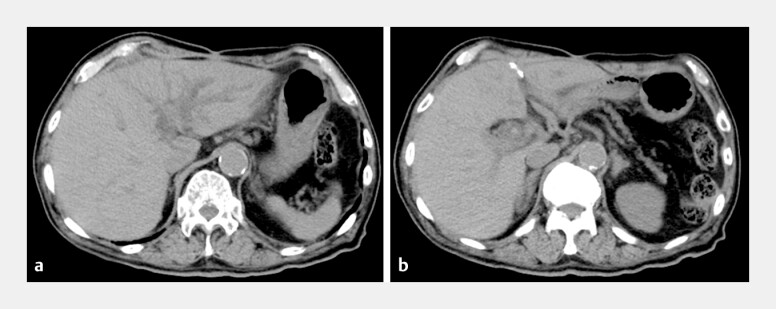
Pre-endoscopic computed tomographic scans. **a** Marked dilatation of the intrahepatic bile ducts, predominantly in the left hepatic lobe, suggesting biliary obstruction due to hepaticojejunostomy strictures. **b** Multiple hyperdense foci consistent with intrahepatic stones are identified within the dilated bile ducts at the hepatic hilum.

**Fig. 2:**
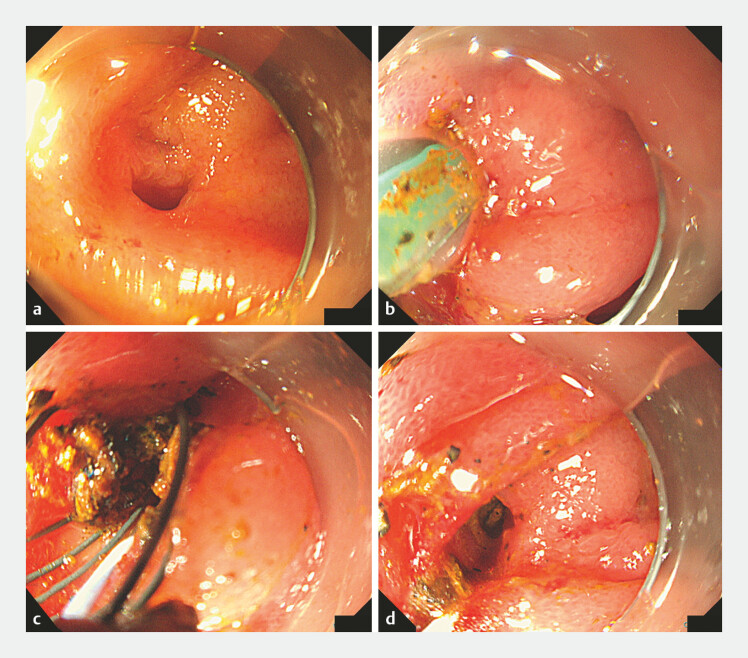
Endoscopic images during stone extraction. **a** Before intervention, two orifices were identified at the hepaticojejunostomy site: the 12 o’clock direction leading to the left hepatic duct and the 6 o’clock direction leading to the right hepatic duct. As significant stones were present in the left intrahepatic duct, stone removal was initiated from this side. **b** Balloon dilation of the anastomotic orifice was performed using a biliary expansion balloon. **c** Stones were retrieved from the left intrahepatic duct using a retrieval basket (RASEN, Kaneka, Japan). **d** Complete clearance of the left intrahepatic duct was achieved; however, the right duct could no longer be visualized due to mucosal changes after repeated intervention. Notably, selective access to the right hepatic duct could not be achieved using conventional ERCP cannulas and guidewires. ERCP, endoscopic retrograde cholangiopancreatography.

**Fig. 3:**
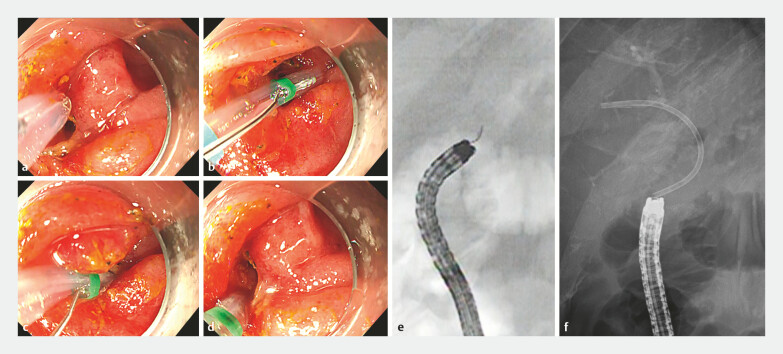
Endoscopic and fluoroscopic images during stone extraction using the novel endoscopic retrograde cholangiopancreatography (ERCP) cannula (Engetsu, Kaneka, Japan). **a** The left hepatic duct orifice was visible, but the right hepatic duct could not be identified; therefore, cannulation into the right intrahepatic duct was initiated using the novel ERCP cannula. **b, c** By rotating the novel ERCP cannula, selective access to the right hepatic duct was attempted under steep anatomical alignment. **d** By sharply bending the device tip along the X-axis toward the 6 o’clock direction, safe and rapid cannulation of the right hepatic duct was achieved. **e** A fluoroscopic image showing the novel ERCP cannula accessing the right hepatic duct in the opposite direction to the endoscope’s natural curvature, highlighting its unique range of movements. **f** Final placement of a plastic stent into the right hepatic duct was successfully performed, confirming the feasibility of selective biliary access in the surgically altered anatomy with acute angulation.

An 85-year-old man who had undergone pancreaticoduodenectomy a year earlier for pancreatic cancer developed recurrent cholangitis. Endoscopic evaluation using SBE ( Fig. 1 ) identified hepaticojejunostomy sites for both the right and the left HBD (L-HBD). The L-HBD was markedly narrowed and contained intrahepatic stones. ERCP cannulation and guidewire insertion into the L-HBD were successfully performed, and cholangiography confirmed the stricture and stones. Dilation with a biliary balloon (REN 8 mm, Kaneka, Japan) followed by stone extraction using a retrieval basket was achieved. Subsequently, we attempted to access the R-HBD; however, after the left-sided procedure, the access angle from the jejunal limb to the R-HBD became sharply angulated, making conventional cannulation difficult. To overcome this, we utilized a novel ERCP cannula (Engetsu, Kaneka, Japan), with 360° rotational control and a wide vertical range of movement along the X-axis 2 3 4 . These features enabled fine adjustments and successful selective cannulation of the R-HBD, despite the steep anatomy. The procedure concluded with the placement of a plastic and metal stent in the R-HBD and L-HBD, respectively, considering the recurrent cholangitis likely caused by anastomotic strictures and the prolonged left-sided intervention time ( Fig. 2 , Fig. 3 , Video 1 ).

Overcoming sharp angulation for biliary access using a novel endoscopic retrograde cholangiopancreatography cannula in the surgically altered anatomy. This video demonstrates the cannula’s technical advantages and successful selective access where conventional devices failed.Video 1

This case demonstrates that the novel ERCP cannula facilitates precise biliary access in the SAA with sharp angulations, where conventional devices often fail. This video highlights its mechanical advantages and potential clinical utility in managing anatomically complex biliary reconstructions.

Endoscopy_UCTN_Code_TTT_1AR_2AK
